# Acid Phosphate

**Published:** 1885-03

**Authors:** 


**HORSFORD'S ACID PHOSPHATE.**

THE LEADING AUXILIARY.

Dr. R. M. Swander, St Louis, Mo., says; "Were I to give a a full report from my note book of the results obtained by the use of Horsford's Acid Phosphate at my instance, it woul l furnish manuscripts sufficient for a large pamphlet; suffice it to say, that my faith in it from practical experience upon myself and many patients, representing a large class of diseases in which the use of phosphorus is indicated, has been of such happy termination that I make it the leading auxiliary wherever the slightest indication for its use presents itself. I should lose confidence in the potency of many of my old favorite formulas did I not feel assured from past observation that my efforts were materially aided by combining the Acid Phosphate therewith, results thus proving."

